# A Trade-off between the Fitness Cost of Functional Integrases and Long-term Stability of Integrons

**DOI:** 10.1371/journal.ppat.1003043

**Published:** 2012-11-29

**Authors:** Irina Starikova, Klaus Harms, Pål Haugen, Tracy T. M. Lunde, Raul Primicerio, Ørjan Samuelsen, Kaare M. Nielsen, Pål J. Johnsen

**Affiliations:** 1 Department of Pharmacy, Faculty of Health Sciences, University of Tromsø, Tromsø, Norway; 2 Reference Centre for Detection of Antimicrobial Resistance, Department of Microbiology and Infection Control, University Hospital of North Norway, Tromsø, Norway; 3 GenØk, Center for Biosafety, Research Park, Tromsø, Norway; University of Oxford, United Kingdom

## Abstract

Horizontal gene transfer (HGT) plays a major role in bacterial microevolution as evident from the rapid emergence and spread of antimicrobial drug resistance. Few studies have however addressed the population dynamics of newly imported genetic elements after HGT. Here, we show that newly acquired class-1 integrons from *Salmonella enterica* serovar Typhimurium and *Acinetobacter baumannii*, free of associated transposable elements, strongly reduce host fitness in *Acinetobacter baylyi*. Insertional inactivation of the integron *intI1* restored fitness, demonstrating that the observed fitness costs were due to the presence of an active integrase. The biological cost of harboring class-1 integrons was rapidly reduced during serial transfers due to *intI1* frameshift mutations leading to inactivated integrases. We use a mathematical model to explore the conditions where integrons with functional integrases are maintained and conclude that environmental fluctuations and episodic selection is necessary for the maintenance of functional integrases. Taken together, the presented data suggest a trade-off between the ability to capture gene cassettes and long-term stability of integrons and provide an explanation for the frequent observation of inactive integron-integrases in bacterial populations.

## Author Summary

Horizontal acquisition of mobile and mobilizable genetic elements plays a major role in the development of antimicrobial drug resistance in bacteria. Despite their causal role in drug treatment failure, there is only limited understanding of how horizontal acquisitions of these elements affect bacterial fitness. A prominent group of such genetic elements are the integrons. These genetic elements harbor an integrase-gene that allows the integron to respond to environmental changes by capture and excision of gene cassettes. Here, we have experimentally determined if horizontal acquisition of an integron affect host fitness. The data demonstrate that the initial costs are substantial. However, inactivation of the integrase gene occurred rapidly by spontaneous mutation alleviating the detrimental effect of the integron on bacterial fitness. The same fitness restoring effects was also shown by targeted inactivation of the integrase gene. The inactivation results in a negative trade-off between host adaptation and loss of the ability to capture new gene cassettes. Importantly, our results explain the frequent observation of inactive integrase genes in integrons found in bacteria of different origins. Finally, we use mathematical modeling to determine the conditions necessary for maintaining functional integrases.

## Introduction

Horizontal gene transfer (HGT) enables bacteria to obtain alien genes and genetic elements from prokaryotic, archaeal, and eukaryotic organisms. This capacity for genetic exchange plays an important role in bacterial adaptive evolution, as exemplified by the rapid spread of antibiotic resistance determinants by HGT [1], [2]. Most often, the fitness effects of novel genes in new hosts are selectively neutral or detrimental [3], and prolonged persistence in the population requires compensatory evolution or associated linked selection [4], [5], [6], [7]. Antibiotic resistance determinants are frequently associated with mobile and mobilizable genetic elements, and they tend to reduce host fitness when newly acquired as part of mobile DNA [4], [5], [8], [9]. The magnitude of these fitness costs as well as the mode and speed of compensatory evolution are key parameters determining the frequency of resistance in bacterial populations following relaxed antibiotic selection (i.e. following interventions on drug prescription levels) [10]. From the perspective of horizontal dissemination of antibiotic resistance determinants, population dynamic studies are important to increase our insight on the evolution and reversibility of resistance [10], [11]. Several studies have described compensatory evolution and host adaptation to self-replicating plasmids [for a selection see [4], [5], [8], [9]]. However, only few studies have considered how bacteria adapt to the presence of chromosomally transferred genes and genetic elements. These studies have been limited to chromosomal allelic replacements [6], [12], transposons [13], [14] and a report on conjugative transposons [15].

Integrons are a class of genetic elements frequently involved in antimicrobial resistance dissemination where population dynamic studies are currently absent. These genetic elements have the ability to capture and excise functional gene cassettes involved in host adaptation, often including antibiotic resistance traits [16]. Typically, an integron consists of an integrase gene (*intI*) encoding a site-specific recombinase responsible for the recruitment and excision of gene cassettes and a promoter (P_C_) for the expression of captured gene cassettes. Integrases capture gene cassettes through recombination between *attI* (located downstream of P_C_) and the gene cassette-borne recombination site *attC* present in a circular gene cassette. Inverse correlations exist between gene-cassette promoter (P_C_) strength and integrase activity [17], [18] as well as expression levels [19]. Based on sequence similarity of the *intI* gene, five classes of “mobile integrons” have been described, for a review see [20].

Class-1 integrons are prevalent in Gram-negative clinical isolates, and harbor gene cassettes encoding resistance to the majority of clinically relevant antibiotics such as aminoglycosides, trimethoprim, and broad-spectrum β-lactams [20], [21]. Structurally, class-1 integrons are relatively diverse, but they generally consist of a 5′-conserved segment (5′-CS) including *intI1*, *attI1*, the variable regions where the gene cassettes are embedded, and a 3′-CS that includes a truncated *qacE1* and *sul1* [22]. Class-1 integrons are frequently linked to complete and incomplete transposons such as Tn*402* [23], and Tn*21*-like structures [24]. Due to the often incomplete nature of the transposable elements linked to clinical class-1 integrons these structures are generally thought to be defective in terms of transposition, and for these elements to move, transposition functions need to be provided in *trans*. However, in clinical isolates, these integron-containing transposons are frequently located on plasmids and thus can easily spread horizontally [25], [26].

Integrons can be important factors for horizontal dissemination of novel and adaptive traits among bacteria because they facilitate “sampling” of the environmental gene-cassette-pool [27], [28]. Moreover the ability to acquire novel cassettes, or shuffle the existing ones, has shown to be increased as a response to stress [29]. Integrons with non-functional integrases are however prevalent in bacterial populations [28], [30], suggesting that the ability to acquire gene cassettes does not necessarily provide a frequent selective advantage. Thus, whereas it is clear that selection for integron-encoded traits such as antibiotic resistance determine the frequency of class-1 integrons in bacterial populations, the selection for functional integrases remains unclear.

Here we show that horizontally transferred class-1 integrons from *Salmonella enterica* serovar Typhimurium and *Acinetobacter baumannii*, free of associated transposable elements, strongly reduce host fitness in *Acinetobacter baylyi*. We demonstrate that these fitness costs are due to an active integrase IntI1. These fitness costs were reduced during serial transfer experiments through mutational inactivation of the integrase gene, suggesting a trade-off between maintaining a functional integrase and stability of integrons in the population over time. Our results provide a rationale for why inactivated integron-integrases are frequently observed in clinical and environmental bacterial isolates. We use a mathematical model to explore the population dynamics of integrons with functional and non-functional integrases in competition with integron-free bacterial populations. We conclude that selection for pre-existing gene-cassettes acts synergistically with the ability to capture new ones [episodic selection [31]] in fluctuating environments.

## Results

### Newly acquired class 1 integrons of reduce fitness in *A. baylyi*

The model organism *A. baylyi* ADP1 is a close relative to the nosocomial pathogen *A. baumannii* and is free of integrons [32]. We constructed a set of *A. baylyi* strains containing cloned diverse class-1 integrons from isolates of two *A. baumannii* (clinical isolates) and one *S. enterica* serovar Typhimurium (isolated from pork). These strains allowed the investigation of the effects of newly acquired integrons on host fitness. The three integrons were inserted in an identical chromosomal locus (ACIAD3309) [32].

**Figure 1:**
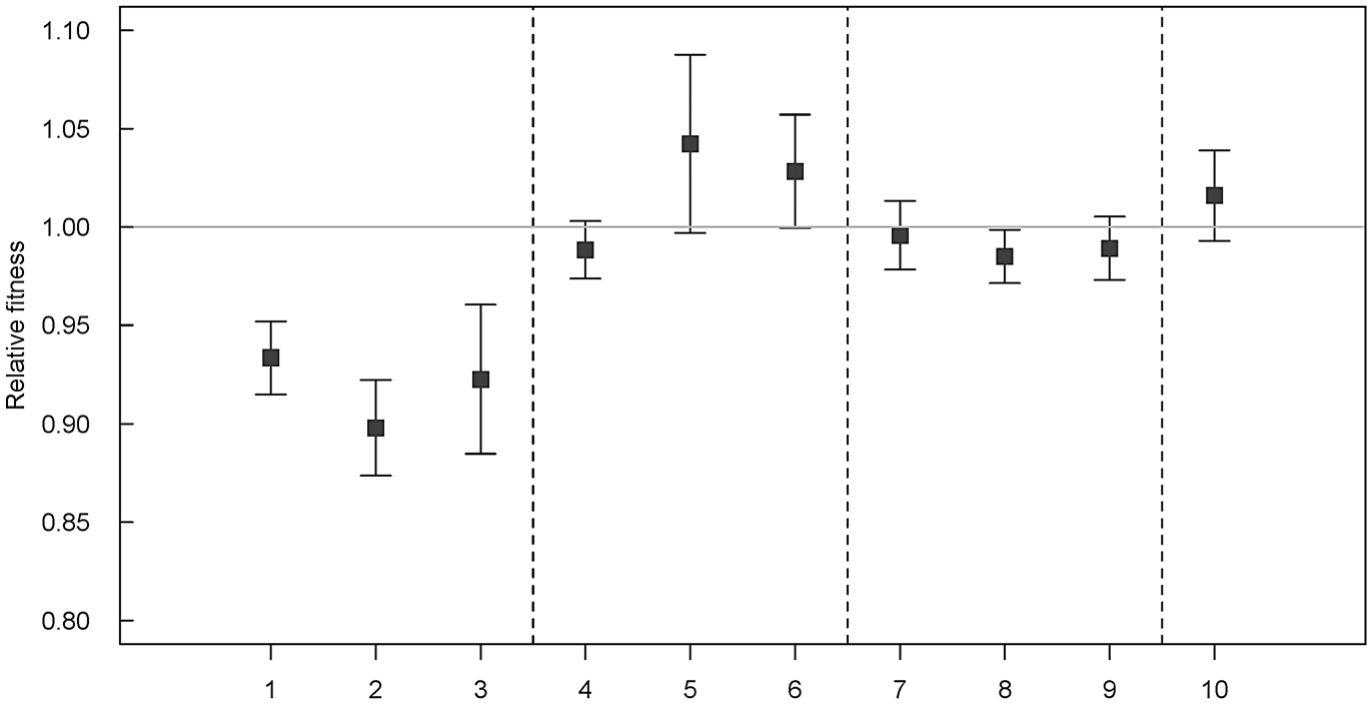
Results from pair-wise mixed culture competition experiments. The integron free *A. baylyi* ADP1 was competed against integron-containing strains with functional or non-functional integrases. Except from the inserted DNA sequences (integrons or *nptII sacB*) in the selectively neutral ACIAD3309 locus, the strains were isogenic. Results were obtained from at least two independent experiments, and number of parallels ranged from 12–50. Error bars indicate 95% confidence intervals. By definition, a relative fitness of 1.0 indicates no difference in relative fitness. Numbers 1–10 on x-axis describes *A. baylyi* ADP1 competed against: 1–3; ADP1 with newly acquired integrons, 4–6; ADP1 with newly acquired integrons insertionally inactivated, 7–9; ADP1 with evolved integrons, and 10 insertion-locus control: 1: IVS1 (w = 0.93 [0.91–0.95]; 2; IVS2 (w = 0.98 [0.97–0.99]); 3: IVS3 (w = 0.92 [0.88–0.96]); 4: IVS1 *intI1*::*cat* (w = 0.98 [0.97–0.99]); 5: IVS2 *intI1*::*nptII sacB* (w = 1.04 [1.00–1.08]); 6: IVS3 *intI1*::*nptII sacB* (w = 1.03 [1.00–1.06]); 7: IVS1_EV-1_ (w = 0.99[0.97–1.01]); 8: IVS1_EV-2_ (w = 0.98 [0.97–0.99]); 9: IVS1_EV-3_ (w = 0.99 [0.97–1.01]); 10: IVS4 (w = 1.01 [0.99–1.03]).

Mixed culture competition experiments revealed that newly acquired class-1 integrons from *A. baumannii* (IVS1 and IVS3) and *S. enterica* serovar Typhimurium (IVS2) resulted in a statistically significant reduced relative fitness (w) of 0.93 (*p* = 0.01**), 0.92 (*p* = 0.02**), and 0.89 (*p*<0.01**), respectively. The relative fitness of the ancestor was by default set to 1.0. The neutrality of the insertion locus (ACIAD3309) was confirmed using a pair of *A. baylyi* ADP1 strains that were isogenic except from the insertion of a selective/counter-selective marker pair in strain IVS4 (*A. baylyi* ADP1 ACIAD3309::*nptII sacB*) (relative fitness w = 1.01, not significantly different from 1.0 (*p* = 0.2)). The results are summarized in Figure 1. To verify that the relative fitness measurements were not hampered by the choice of selective antibiotic resistance markers all fitness measurements presented in this study were repeated with strain IVS4 as an integron-free competitor. The results from these experiments using sucrose (*sacB*) counter-selection were always congruent with kanamycin, spectinomycin, and spectinomycin/ampicillin selective platings, and the results from all parallel competition experiments were pooled before statistical analyzes.

### Fitness costs of class-1 integrons with strong and weak cassette promoters (P_C_)

The class-1 integrons inserted into *A. baylyi* ADP1 differed in their gene cassette promoter sequences, located in the *intI1* open reading frame. Sequence alignments of the three *intI1* sequences inserted into *A. baylyi* revealed that the integrons with the highest (IVS2), and lowest (IVS1) fitness costs contained cassette promoters identical to the recently described weak (P_C_W) and strong (P_C_S) promoters, respectively [17]. The difference in relative fitness between strains IVS1 and IVS2 was statistically significant in independent sample t-tests (*p* = 0.03*), suggesting a correlation between integrase activity and the fitness cost of harboring an integron. The integron with the intermediate fitness cost (strain IVS3, w = 0.92±0.04) contained a hybrid P_C_ promoter.

### Inactivation of the integrase gene restored fitness

In three integron-containing *A. baylyi* strains (IVS1, IVS2, and IVS3), the *intI1* integrase genes were inactivated by insertions of either *cat* (strain IVS1 *intI1*::*cat*) or *nptII sacB* cassettes (strains IVS2 *intI1*::*nptII sacB* and IVS3 *intI1*::*nptII sacB*). These *intI1* knockout mutants displayed no significant reduction in relative fitness in mixed competition experiments with the ancestral *A. baylyi* ADP1 (Figure 1). To test the hypothesis that strains with inactivated integrases increased fitness when compared to their functional counterparts, independent sample t-tests were performed. For all pairs, the *intI1* inactivation restored fitness completely: IVS1 vs. IVS1 *intI1*::*cat* (p = 0.015**), IVS2 vs. IVS2 *intI1*::*nptII sacB* (p<0,001**), and IVS3 vs. IVS3 *intI1*::*nptII sacB* (p = 0,003**). These data further demonstrate that the initial fitness cost of integron-carriage was due to the presence of an active integrase. Expression of the integrase genes in the chromosomal insertion locus was verified by reverse transcription PCRs (RT-PCR) in IVS1, IVS2, and IVS3. No transcripts were detected in strains IVS1 *intI1*::*cat*, IVS2 *intI1*::*nptII sacB*, and IVS3 *intI1*::*nptII sacB* (Figure S1).

### Inactivated integrase genes emerged during serial transfer experiments

A total of 20 *A. baylyi* IVS1 cultures were subjected to daily 1∶100 dilutions in fresh LB medium. During the serial transfer experiments the evolving populations were screened for colonies of increased size on LB agar plates, a method regularly used to identify fitness compensated mutants [33], [34]. Twice a week agar plates were visually inspected and the first colony of increased size appeared after 30 days in one of the populations. This colony was isolated and frozen down for further analyses. At day 42 we isolated two additional colonies from different populations. These isolates were analyzed and they all contained mutations in the *intI1*. Complete integrons from these three evolved *A. baylyi* IVS1 genetic backgrounds were transferred back into the ancestral *A. baylyi* ADP1 strain, yielding strains IVS1_EV-1_, IVS1_EV-2_, and IVS1_EV-3_.

To test the hypothesis that the evolved integrons increased fitness, they were competed against the ancestral ADP1. Mixed culture competition experiments revealed that fitness was completely restored in these strains (Figure 1). Independent sample t-tests further verified that the relative fitness of the each evolved integron was significantly different from its *intI1-*functional ancestor IVS1, (p = 0,001**, for all three comparisons). Subsequent characterizations of these three transformants by DNA sequencing revealed frameshift mutations close to the start codon of the *intI1* open reading frame rendering the integrase non-functional (Figure S2). RT-PCR of evolved strain IVS1_EV-1_ yielded no transcript (Figure S1).

### Theoretical results

**Figure 2:**
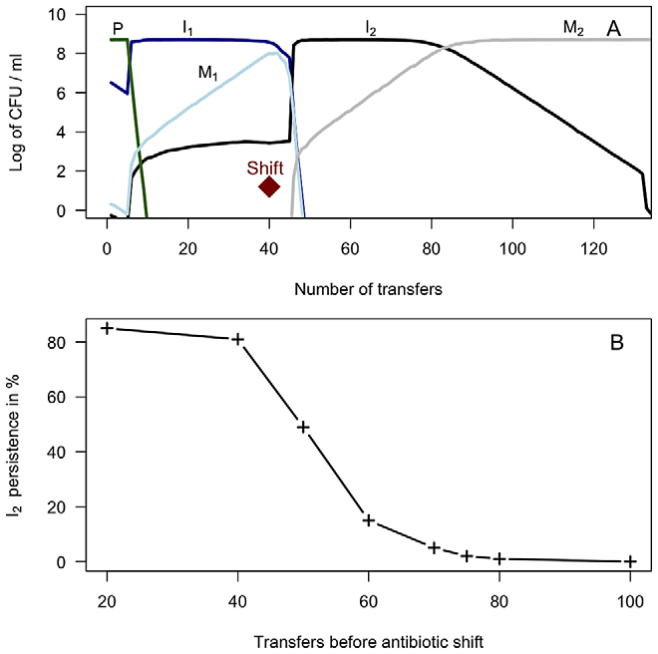
2A) Simulation results depicting the dynamics of integron-containing and - free populations driven by competition and antibiotic selection in serial transfer cultures. Diamond indicates antibiotic switch. Population I harbors an integron with a functional integrase with one gene cassette encoding resistance to antibiotic A (dark blue line). Population I_2_ has acquired a second gene cassette and encodes resistance to both antibiotics A and B (black line). Following frameshift and nonsense mutations in the functional integrase, populations I_1_ and I_2_ form M_1_ (light blue line) and M_2_ (grey line), respectively. Population P (green line) is the integron-free wild type. The results shown are the median values of 100 realizations until the I_2_ population falls below 1 CFU per ml. 2B) Persistence of integrons with functional integrases: The connected crosshairs presented in figure 2B shows the proportion of realizations where the functional integrase (in population I_2_) is still present after 50 additional transfers following different time intervals between shifts from antibiotic A to B.

We hypothesized that functional integrases are maintained by episodic selection provided by fluctuating environments [31]. To test this hypothesis *in silico* we parameterized a mathematical model with our own experimental data, and relevant parameters from the literature. Parameters related to resource utilization (*e* and *km*) were calibrated to yield population sizes close to what we observed in the laboratory. The MIC values were based on our own experiments, parameters on growth characteristics were derived from our own study (fitness values) combined with values from the literature. For a complete list of parameters used in these serial transfer simulations, see Table 1. Fig. 2A shows the predicted population dynamics of strains harboring a newly acquired integron with a functional integrase with one (I_1_ – blue line) and two (I_2_ – black line) gene cassettes, the integron free susceptible wild type (P – green line), and two fitness ameliorated integrase- mutants (M_1_ - light blue and M_2_ - grey). The predicted *in silico* population dynamics, before “shift” in Figure 2A, mirrors our experimental data form the serial transfer cultures where integrase specific fitness compensating mutants were isolated after 30 and 42 days of serial transfers. These single mutants were selected on antibiotic free agar plates with approximately 100 colonies, suggesting an approximate frequency of 1/100. Fluctuating environments are simulated by a probability of encountering antibiotic A for a period of 40 transfers, and then antibiotic B for the remaining time period, both at a 10% probability per transfer. The results shown in Fig. 2A are the median values for 100 simulations. Our simulations show that functional integrases are descending when only one antibiotic is present. However, the switch to a second antibiotic B allows the pre-existing two-gene cassette integron (I_2_) to rapidly ascend to high frequency. During this ascent I_1_ and M_1_ are driven extinct. Without further environmental change, the mutated integrase M_2_ outcompetes its less fit counterpart I_2_. As shown in Fig. 2B, persistence of integrons with functional integrases strongly depends on when the switch to antibiotic B occurs. To assess the robustness of the model predictions scenario in Fig. 2A we explored different parameter ranges for the gene cassette acquisition rate (λ), mutation rate for inactivated integrase (π), and the mutation rate for restoration of functional integrase (θ). We performed 500 additional simulations where π and θ were varied over 10 values each, and λ over 5 levels (ranges provided in Table 1). As illustrated in Figure S3 the model predictions were robust for a wide range of these parameter combinations. Further, we explored the extreme values of the 95% CI of the relative fitness parameter V as experimentally determined (w = 0.91 and 0.95) alone and in combinations with different parameter values. These values and the mean fitness value (w = 0.93) for V_I_ were tested when π, θ, *and* λ varied over a small range (±.2.5%) to assess changes in model predictions. Qualitatively all additional simulations *(n = 581)* were consistent with the scenario presented in Figure 2A providing generality to the model predictions (data not shown).

**Table 1:** Mathematical model and simulations: list of parameters.

Parameter	Description	Value	Source
**Parameters for growth rate (Φ)**			
V_x_	Maximum growth rate	X = I_1_, I_2_: V = 0.93, (0.91,0.95) X = P, M_1_, M_2_: V = 1	This study
U_x_	Maximum kill rate	X = P: U = −0.01, All others U = −0.2	[31]
MIC_x_	Minimum Inhibitory Concentration	**For Antibiotic A**	This study
		X = I_1_, I_2_, M_1_and M_2_: MIC = 24 g/L X = P: MIC = 0.5 g/L	
		**For Antibiotic B**	
		X = I_1_, P, M_1_: MIC = 0.1 g/L X = I_2_, M_2_: MIC = 16 g/L	
k	Hill coefficient	1.0	***
km	Resource concentration where 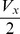	0.25 g/L	[31]
**Parameters for differential equations**			
e	Conversion efficiency of resources	1.0×10^−7^	[57]
θ	Mutation rate, restoration of functional integrases	7.5×10^−11^ bp^−1^generation^−1^ (1.0×10^−13^ to 1.0×10^−8.5^)	[58]
π	Mutation rate for generating defective integrases	3.75×10^−8^ generation^−1^ (θ×500) * (1.0×10^−13^ to 1.0×10^−8.5^)×500	[58]
λ	Rate of gene cassette acquisition	1.0×10^−8^ generation^−1^ (1.0×10^−9^ to 1.0×10^−5^)	**
da/db	Decay rates of antibiotics A and B	0.05	***

* We assume that nonsense and frameshift mutations render the integrase non-functional if it occurs within the first 500 bp of the integrase.

** Gene cassette acquisition rates were conservatively set at least two orders of magnitude lower than low conduction frequencies as reported for example in [59].

*** Hill- coefficient arbitrarily set to 1 [within the range presented in [49]] and decay rates were adjusted to ensure efficient antibiotic selection. Values in brackets for V_x_, θ, π, and λ are the widened parameter ranges applied in model predictions.

## Discussion

We show for the first time that newly acquired integrons can substantially reduce relative fitness of its new bacterial host. Following the insertion in a selectively neutral chromosomal locus, the three class-1 integrons from isolates of *A. baumannii* and *S. enterica* serovar Typhimurium reduced fitness in the *A. baylyi* recipient by 7–11%. For comparison, these fitness costs are in the range of mutations conferring antimicrobial resistance through modifications of housekeeping genes such as *par/gyr* mutations (fluoroquinolone resistance) in *Streptococcus pneumoniae* [35], and some *rpoB* mutations in *E. coli* [36]. Direct insertional inactivation of the three *intI1* alleles completely mitigated the initial fitness reductions, clearly suggesting that the fitness costs observed were due to the presence of a functional integrase gene (*intI1*).

Non-functional integrase genes due to frameshift- and nonsense-mutations are frequently encountered in surveys [28], [30], [37]. We asked whether functional *intI1* genes would be inactivated during experimental evolution. After 30–42 days of daily serial transfers we observed colonies of increased size on agar plates, representing putative fitness compensated mutants. Integrons from evolved isolates were subsequently introduced into the ancestral genetic *A. baylyi* ADP1 background, and in these strains they no longer reduced fitness of the host bacterium (Figure 1). Sequence analyses of the three *intI1* genes revealed the presence of frameshift mutations in the first quarter of *intI1* resulting in premature stop codons, rendering these integrases inactive (Figure S2). The emergence of non-functional *intI1* genes during experimental evolution with mutational inactivation patterns identical to those reported from bacterial isolates of environmental and clinical origins [28], [30], [37] strongly suggests that integrase pseudogenes may ascend to high frequencies in bacterial populations by natural selection.

It was recently demonstrated that *intI* expression is under the control of the SOS response through the presence of LexA binding sites in the integrase promoters (including class-1 *intI1*) [29], [37]. These authors proposed that LexA repression reduce the potential detrimental effects of *intI* expression, and that SOS induction allows expression of the integrase gene when new gene cassettes could provide a response to stressful and potentially lethal environmental conditions [29], [37]. It was also suggested that integrase inactivation is correlated with absence of LexA regulation [37], and that this is a key factor explaining the high proportion of pseudo-*intI*-genes found in integron-containing bacteria [28], [30], [37]. The experimental data reported here are the first to support both these hypotheses. The majority of *Acinetobacter* species, including our model organism *A. baylyi* and the clinically relevant *A. baumannii* all lack *lexA* homologues [38], [39]. Thus, *intI1* is most likely not under LexA repression in our model system, and the newly acquired integrons reduced fitness in *A. baylyi*, despite the presence of native LexA binding sites in two out of three integrons. The mutational inactivation of *intI1* completely mitigated the fitness costs of integron carriage, and in the absence of repression the inactivation could very well mimic tight repression of integrase expression.

The serial transfer experiments were performed in nutrient-rich LB medium, as opposed to minimal medium for the competition experiments. The emergence of fitness compensated *A. baylyi* with non-functional integrases during experimental evolution strongly suggests that the fitness costs of integron carriage are not limited to specific growth conditions. Consequently, the fitness restoration due to *intI1* inactivation leads to stabilization of the cassette arrays in the bacterial population, and integron-borne antibiotic resistance determinants will not be reduced following relaxed selective antibiotic pressures.

Previous reports indicate an inverse correlation between gene-cassette promoter (P_C_) strength and integrase activity [17], [18] as well as expression levels [19]. From the results presented in these reports it could be hypothesized that a strong gene-cassette promoter would decrease the overall activity of the integrase gene, and that the cost of integron carriage should be reduced. Our results favor this hypothesis. However, the results should be interpreted with some caution since we achieved significance at the alpha level, but not when Bonferroni correction was applied. The newly acquired integron from a clinical *A. baumannii* strain (∼7% fitness cost) contained a cassette promoter sequence identical to the “strong promoter” (P_C_S) whereas the cassette promoter of the integron from the *S. enterica* serovar Typhimurium strain (∼11% fitness cost) displayed a “weak promoter” (P_C_W), as reported by Jove *et al.* [17]. Moreover, the integrase sequence from *S. enterica* serovar Typhimurium revealed amino acids in positions 32 (R), and 39 (H) consistent with the highest recombination activity demonstrated in [17]. Jove and co-workers suggested that increased expression of gene cassettes, leading to higher levels of resistance, would be selected for in environments with strong antibiotic selective pressures. Our results add complexity to that hypothesis insofar that the increased expression of gene-cassettes also could lead to reduced integrase activity, and thus stabilize functional integrons in non-selective environments.

Two lines of evidence support that the mechanistic basis for the observed fitness effects of functional integrases is reduced genomic stability. First, IntI1 can catalyze recombination events between *attI*/*attC* sites and frequently encountered non-canonical sites in the genome, as demonstrated by Recchia and co-workers [40]. Secondly, purified IntI1 enzyme possesses all functions necessary for target recognition and recombination, as shown in *in vitro* strand transfer assays [41], [42]. Consequently, when newly acquired and in the absence of tight regulation, expressed integrase would be able to form recombination junctions between the integron and sequence-regions elsewhere in the genome. Resolution of such single strand crossovers ultimately leads to potentially lethal deletions of the genomic region between the recombination sites either following replication or IntI1 activity, as demonstrated in co-integrate resolution experiments [40].

We hypothesized that environmental fluctuations and episodic selection [31] are key to the maintenance of functional integrases, and explored this in computer simulations. According to our hypothesis selection for pre-existing gene cassettes in integrons (type-1 episodes) acts synergistically with the ability to capture new cassettes that provide bacteria with a selective benefit in changing environments (type-2 episodes). Type-1 episodes favoring pre-existing gene cassettes allow integrons to reach high frequencies in the population but during these conditions, due to the fitness cost of the active integrases, non-functional integrases rapidly ascend in the population. Type-2 episodes select for new gene cassettes acquired by the active integrase. Our simulations show that maintenance of functional integrases depends on the time between the different episodes (i.e. the frequency of environmental change), as well as the continuous availability of new and adequate gene cassettes. Of course the selective episodes could be other favorable traits encoded by gene-cassettes, and are not limited to antibiotic resistance determinants.

In conclusion, the presented data suggest that in the absence of *intI1* repression, a fitness trade-off exists for the maintenance of integrons with functional integrases. The initial high fitness cost of the integrase can only be outweighed by selection for gene cassette dynamics.

## Materials and Methods

The bacterial strains, plasmids, and primers used in this study are listed in Tables 2, S1 in Text S1, and S2 in Text S1. Strains were grown in S2-minimal medium, amended with 2% lactate [43], or Luria Bertani (LB) agar or broth at 30°C or 37°C under aeration.

**Table 2:** The strains used in this study; genotypes and relative fitness.

*A. baylyi* strains	Genotype, origins of integrons, and description^1^
IVS1	Integron from *A. baumannii* 064, clinical isolate [55]: [*intI1*|*aadB*|*qacEΔ1*|*sull1*|*orf5*]
IVS1 *intI1*::*cat*	IVS1 with inactivated integrase: [*intI1*::*cat*|*aadB*|*qacEΔ1*|*sull1*|*orf5*]
IVS1_EV-1_	ADP1 with evolved IVS1 integron 1: [*intI1′*|*aadB*|*qacEΔ1*|*sull1*|*orf5*]
IVS1 _EV-2_	ADP1 with evolved IVS1 integron 2: [*intI1″*|*aadB*|*qacEΔ1*|*sull1*|*orf5*]
IVS1 _EV-3_	ADP1 with evolved IVS1 integron 3: [*intI1′″*|*aadB*|*qacEΔ1*|*sull1*|*orf5*]
IVS2	Integron from *S. enterica-* Serovar –Thyphimurium- 490 – food isolate [55]: [*intI1*|*bla*_oxa30_|*aadA1*|*qacEΔ1*|*sull1*|*orf5*]
IVS2 *intI1*::*nptII sacB*	IVS2 with inactivated integrase: [*intI1*::*nptII sacB*| *bla*_oxa30_ |*aadA1*|*qacEΔ1*|*sull1*|*orf5*]
IVS3	Integron from *A. baumannii*- 47-42 [56]: [*intI1*|*aacC1*|*orfP/P/Q*|*aadA1*|*qacEΔ1*|*sull1*|*orf5*]
IVS3 *intI1*::*nptII sacB*	[*intI1*::*nptII-sacB*|*aacC1*|*orfP/P/Q*|*aadA1*|*qacEΔ1*|*sull1*|*orf5*]
IVS4	(*nptII sacB*)

1 Integrons were all inserted in *A. baylyi* locus ACIAD3309.

### Plasmid constructions

Plasmid pTM4 is derived from the pGT41 [44] and was used for *in vitro* insertion of integrons into a chromosomal locus. pTM4 contains segments identical to upstream and downstream segments of the 5′-region of the chromosomal *A. baylyi* ACIAD3309 open reading frame for homologous recombination, interrupted by a *Sac*I/*Ecl*136II restriction site, and was constructed as follows: The downstream segment (707 bp) was PCR-amplified with primers ACIAD3309-down-F (including a 5′-heterologous tail containing an *Ecl*136II/*Sac*I site) and ACIAD3309-down-R (Table S2, in Text S1) with Phusion DNA polymerase (Finnzymes, Espoo, Finland) according to the manufacturer's instructions but with 10% DMSO added, and inserted into the *Ksp*AI site of pGT41, giving pTM1. The upstream segment (785 bp) was amplified with primers ACIAD3309-up-f and ACIAD3309-up-r (with 5′-*Ecl*136II/*Sac*I tail) and inserted into the *Oli*I site of pTM1, giving pTM2. From pTM2, two unwanted segments were removed as follows: A 2.7 kbp insert containing an *nptII* (kanamycin resistance) gene was excised by cleavage with *Sac*I (has 2 sites in pTM2) and re-circularization of the large fragment, resulting in pTM3 which has the two segments for homologous recombination ligated immediately upstream and downstream of an *Ecl*136II/*Sac*I restriction site. From pTM3, the *bla* (ampicillin resistance) gene was truncated and rendered non-functional by cleavage with *Xmn*I (contains 2 sites in pTM3) and re-circularization of the large fragment, giving pTM4.

### Construction of class-1 integron containing *A. baylyi*

*A. baylyi* IVS1 was constructed as follows: The integron of *A. baumannii* Ab064 (Table 2) including the 5′- and 3′-CS was PCR-amplified with Phusion polymerase using 5′-phosphorylated primers IntF2 and OrfRev3 (Table S2, in Text S1) and ligated to *Ecl*136II-cleaved (blunt-ended linear) pTM4, respectively. The ligation assay was used as donor DNA to naturally transform (see below) *A. baylyi* ADP1. Transformants were selected on medium containing kanamycin (25 µg/ml). One transformant was generated from a PCR product covalently joined to a vector molecule at both ends and that substituted the 5′-end of ACIAD3309 with the integron from *A. baumannii* Ab064, and termed IVS1. Co-integrates were excluded by screening for chloramphenicol sensibility, and the desired insertion was verified by PCR. The strains IVS2 and IVS3 were constructed as described for IVS1 with integrons of *S. enterica* serovar Typhimurium 490 and *A. baumannii* 47-42 (Table 2), respectively, using primers IntF2/OrfRev2 and employing corresponding selection and PCR controls. The three class-1 integrons differed in the variable regions (Table 2) as well as in the integrase sequences (different gene cassette promoters and SNPs). The integrase accession numbers are JX041889 (*A. baumannii* Ab064), AM991977 (*S. enterica* serovar Typhimurium 490), and JX259274 (*A. baumannii* 47-42). Strain IVS4 (locus neutrality control) was obtained by transformation of *A. baylyi* ADP1 by pTM2 (kanamycin-resistant, sucrose-sensitive, verified by PCR).

### Insertion inactivation of *intI1*

The *intI1* gene of IVS1 was disrupted by natural transformation with *Hin*cII-linearized pACYC177-*int-cat* as substrate for natural transformation (Table S1, in Text S1). This plasmid contains an internal fragment of the *intI1* gene of *A. baumannii* AB064 with a *cat* (chloramphenicol resistance) gene inserted. The resulting strain was PCR-verified and termed IVS1 *intI1*::*cat*. The *intI1* genes of IVS2 and IVS3 were insertion-inactivated in a corresponding manner by pACYC177-*int-nptII-sacB*, which contains the *nptII sacB* marker pair (kanamycin resistance/sucrose susceptibility) from pTM2 (cloned as *Ecl*136II fragment) instead of *cat* [45], [46]. The resulting strains were verified phenotypically, and by PCR and termed IVS2 *intI1*::*nptII sacB* and IVS3 *intI1*::*nptII sacB*, respectively.

### Serial transfer experiments and transfer of evolved integrons back into the ancestral genetic background

Strain IVS1 with a class-1 integron from *A. baumannii* Ab064 was subjected to daily one hundred-fold dilutions in 10 ml LB broth in 20 independent parallels for 30–42 days. Aliquots were plated every third day on LB agar plates to screen for fitness-compensated mutants by increased colony size. Evolved integrons were transferred back into the ancestral *A. baylyi* ADP1 background by PCR-amplification including surrounding regions of homology using homologous transformation (yielding strains IVS1_EV-1_, IVS1_EV-2_, and IVS1_EV-3_) (Table 2).

### Fitness measurements

Integron-containing and -free *A. baylyi* ADP-1, otherwise isogenic, were subjected to mixed competition experiments as previously described [12], [47] with the following modifications: Competing strains were pre-grown in S2 minimal media for 24 hours before diluting 1∶10 in NaCl (0,9%), and 150 µl of each competitor was transferred and mixed into 2.7 ml S2 medium supplied with 0.1% DNase (to exclude natural transformation in the assays). Initial (N_0_) and final densities (N_24_) of competing strains were measured before the onset of competitions and after 24 hours by selective and non-selective plating. Selective traits exploited were antibiotic resistance markers or a counter-selective marker (*nptII* or *aadB*, kanamycin resistance; *aadA*, spectinomycin resistance; *bla*_OXA-30_ ampicillin resistance; *sacB*, sucrose susceptibility). From these densities, the Malthusian parameter (*m*) of each competitor was determined using the equation *m* = ln (N_24_/N_0_). Relative fitness (*w*) was estimated as the ratios of each competitor's Malthusian parameter (*m_1/_m_2_*) [47]. To avoid potential marker-bias *m_1_* and *m_2_* were estimated by selective plating on antibiotics (kanamycin/spectinomycin/ampicillin) in one genetic background followed by sucrose selection in the other. Results were always congruent for the antibiotics and concentrations chosen, and data from both selective regimes were pooled. Estimates of *w* were based on 12–24 parallel experiments for each competition experiment.

### Natural transformation

Preparation of competent cells and transformation assays were performed as described previously [12], [48] with some modifications. Briefly, competent cells were prepared by diluting an overnight culture of *A. baylyi* 1∶100 in fresh LB. The culture was incubated at 30°C with vigorous shaking until the cell titer reached 1×10^9^ ml^−1^. The cells were chilled on ice, pelleted by centrifugation at 5000×g and 4°C for 15 min, and re-suspended in LB supplemented with 20% glycerol. Aliquots were stored at −80°C until use. For transformation, competent cells were thawed on ice and diluted 1∶40 in LB medium containing the donor DNA. The assays were aerated for 90 min at 30°C and plated on selective media plates in appropriate dilutions. The plates were incubated at 30°C until visible colonies had formed (16–40 hours).

### Phenotypic and genotypic characterization

The minimal inhibitory concentrations (MICs) of the donor, recipient and transformant strains were determined for sulfamethoxazole, kanamycin, streptomycin, spectinomycin, gentamicin, and ampicillin, by E-test according to the instructions of the manufacturer (BioMeriux, France). Nucleic acids were isolated with QIAGEN Genomic/Plasmid DNA kits (QIAGEN, Germany), according to the manufacturer's instructions.

The transformation assay using *A. baumannii* 064, *S. enterica* serovar Typhimurium 490 and *A. baumannii* 47-42 as donors, resulted in a number of transformants that were analyzed phenotypically (MIC values, Table S3, in Text S1) and genotypically. Primers IntF2/OrfRev3 and IntF2/OrfRev2 were used to amplify the entire integron region in both transformants and donor strains, giving approximate sizes of 4 kb, 5 kb, and 6 kb for *A. baumannii* 064, *S. thyphimurium* 490 and *A. baumannii* 47-42 transformant strains, respectively. Primers 5CS′/3CS′ were used to verify the size of the variable regions in both donor and test strains; primers UpF/DownR as well as IntF2/3CS′ and 5CS′/OrfRev2/OrfRev3 were used to confirm the correct position of the aquired integrons in the ADP1 genome. Primers IntF2/OXA303R and IntF2/aacC1-OrfP-R were used to verify the position of the gene cassettes within an integron in the strains IVS2 and IVS3, respectively. Primers aadBF/aadBR, OXA305F/OXA303R and aacC1-F2/aacC1-orfP-R (Table S2, in Text S1) were used for gene cassettes identification within the integrons.

The unknown regions surrounding the integron in the donor were sequence determined by direct genomic DNA sequencing (primer walking) as described previously [12] with the following modifications: 20 µl sequencing reactions consisted of 4 µl BigDye v3.1 sequencing mix (Applied Biosystems), 4 µl of the primer at a concentration 10 mM, 4 µl of a sequencing buffer, and ∼4 µg of the purified chromosomal DNA. The sequences of the integrons in the donor strain and transformants were determined by sequencing (BigDye Chemistry) of the PCR products obtained from the primers IntF2/OrfRev3, or IntF2/OrfRev2 (Table S2, in Text S1). The sequence of the integrase gene was determined by sequencing of the PCR products amplified with the primers IntF2/aadBR, IntF2/GCS1RevComp, and INCINTF/IntI1F. PCR products were purified by adding a mix of exonuclease 1 (0.2 U/µl PCR product) (New England Biolabs) and shrimp alkaline phosphatase (0.01 U/µl PCR product) (Roche) followed by 30 minutes incubation at 37°C and 5 minutes at 95°C in a PCR machine. The obtained sequences were analysed by the Sequencher v.4.2.2 programme (GeneCodes, USA) and compared to previously published sequences (GenBank).

### Reverse transcription PCR (RT-PCR)

RNA was isolated using the Total RNA Isolation KIT (Macherey-Nagel, Germany), and cDNA was synthesized using MonsterScript 1^st^-strand cDNA synthesis Kit (Epicentre Biotechnologies, USA), both according to the manufacturer's instructions. The generated cDNA was amplified using primers INCINTF/IntI1F (Table S2, in Text S1).

### Theoretical model

To investigate the conditions that favor maintenance of integrons in bacterial populations, we used a mathematical model and numerical solutions, based on [31] [49]. This serial passage model included five populations. Populations I_1_ and I_2_, contain functional integrases where I_1_ has captured a single cassette encoding resistance to antibiotic A, I_2_ has captured two gene cassettes and is resistant to both antibiotics A, and B. Populations I_1_ and I_2_ can acquire frameshift mutations in *intI1* and turn into populations M_1_ and M_2_ with non-functional integrases, respectively. Population P is the antibiotic susceptible, integron-free wild type. The growth rates of I, M, and P populations are determined by the pharmaco-dynamic function developed by Regoes and co-workers [49], where a Hill-function determines the growth rate or death rate (negative growth rate) of the populations in the presence of antibiotics [50], [51]. Briefly, the growth rate 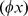 depends on the concentration of resource (R), antibiotics (A and B), and antibiotic susceptibility (MIC). In this model each population have two different growth rates; 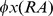 and 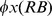. In the simulations 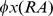 is chosen if antibiotic A is present, and 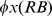 when B is present, such that 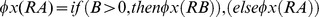. Thus, the model does not simulate events where both antibiotics are present. With these definitions the changes in the population densities during one serial transfer event of I, M, and P populations are given by the following equations:
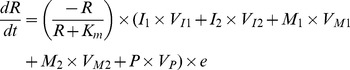


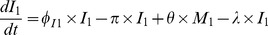


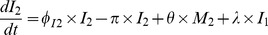


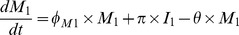


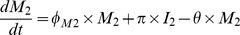


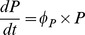


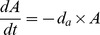


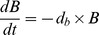
where *e* µg/ml is the conversion efficiency (the resource concentration necessary to produce one new cell) [52], *d_a_* and *d_b_* are the decay rates of the antibiotics, π is the mutation rate for generating defective integrases, and θ is the mutation rate for restoring functionality of defective integrases. λ is the rate at which populations with functional integrases acquire gene cassettes. An illustration of the model with respect to π, θ, λ is given in Figure S4. A list of parameter values is given in Table 2.

Following each simulated dilution (1∶100) 50 µg/ml of the resource was added, and the introductions of antibiotics were stochastic events. Each transfer was assigned a random value (range 0 to 1) from a uniform distribution of numbers. When this value was above a defined probability of 10%, antibiotics were added at 2× (antibiotic A) and 10× (antibiotic B) the MIC concentration of the susceptible populations in order to ensure proper selective effects of the added antibiotics. To investigate the temporal effect of fluctuating environments on the population dynamics of integron containing populations the temporal switch from antibiotic A to B was set at days 20, 40, 50, 60, 70, 75, 80, 100. A total of 100 simulations were performed at each frequency. To qualitatively test the robustness of the model predictions 500 additional simulations were run for different combinations of θ (10), π (10), and λ (5) within the ranges provided in Table 2. We also tested the model behavior where the parameters θ, π and λ were combined with a small variation around the original selected model parameter (±2.5%) for three levels of the relative fitness of integron carriage parameter (V_x_). These levels of V_x_ included the extreme values from the 95% confidence intervals provided in the experimental measurements. For a numerical solution of the differential equations and to simulate the experimental conditions, the open source computer program R version 2.14.1 was used [53]. Dilutions as well as introduction of resource and antibiotics were determined by the events argument in the lsoda function from the deSolve package version 1.10-3 [54].

### Theoretical model, assumptions and parameters

We assume that gene cassettes are available for the populations with functional integrase. Further, the resistance genes are assumed to be selectively neutral, as supported by the experiments conducted in this study. We model the use of two antibiotics to show the principle of a heterogeneous environment and the antibiotics are assumed to have no interactions. In these simulations a cut off was set at 1 CFU per ml where all growth and interactions were stopped. All populations were diluted 1∶100 every 24 hours. For simplicity, gene cassette reshuffling (the order of resistance genes) or loss of single gene cassettes was not considered. For each set of environmental variables the median population densities from 100 simulations were calculated for each time point and the logarithm of the densities plotted at 24-hour intervals until I_2_ population reaches 1 CFU/ml.

### Statistical analyses

Parameter estimation and statistical tests were performed in SPSS vs. 17. In addition to significance at the alpha level (0.05*), multiple testing issues were addressed by Bonferroni corrections of significance levels (indicated as ** throughout the text).

### Accession numbers

*IntI1* from *A. baumannii* Ab064: JX041889.

*IntI1* from *S. enterica* serovar Typhimurium 490: AM991977.

*IntI1* (partial) *A. baumannii* 47-42: JX259274.

## Supporting Information

Figure S1 Upper row: RT-PCR with integrase-specific primers (INCINT/IntI1F) confirms expression of the integrase in the constructed strains IVS1 (lane 2), IVS2 (lane 5) and IVS3 (lane 7); for the strains IVS1 *intI1*::*cat* (lane 3), IVS1_EV-1_ (lane 4), IVS2 *intI1*::*nptII sacB* (lane 6) and IVS3 *intI1*::*nptII sacB* (lane 8) expression of the integrase is below the detection limit. ADP1 was chosen as a negative control for the expression of the integrase (lane 9). RT-PCR does not reveal any amplification with RNA samples in all the strains (lanes 12–19). Lanes 1 and 11- molecular weight marker 1 kb+ DNA-ladder; lanes 10 and 20 - water controls. Bottom row: RT-PCR with 16SrDNA-specific primers (16SF/16SR) was performed to confirm the expression of this gene in all the strains: IVS1, IVS1 *intI1*::*cat*, IVS1_EV-1_, IVS2, IVS2 *intI1*::*nptII sacB*, IVS3, IVS3 *intI1*::*nptII sacB*, ADP1 (lane 2–9, respectively). RT-PCR with RNA samples does not show amplification of the gene (lanes 12–20). Lane 1- molecular weight marker 1 kb+ DNA-ladder; Lanes 10 and 11 - water controls.
(TIFF)

Figure S2 Amino acid sequence of the ancestral-(top), and evolved *intI1* (IVS1_EV-1_, IVS1_EV-2_, IVS1_EV-3_) strains. Amino-acids in bold indicate identity to the ancestral strain. All evolved strains revealed early frameshift mutations: Deletion of G_142_ in IVS1_EV-1_/IVS1_EV-2_. In IVS1_EV-3_ an additional G was inserted in position 98. Consequently, early stop-codons emerged rendering the respective gene products inactive.
(TIFF)

Figure S3 Model predictions for broad ranges of the parameters λ (gene-cassette acquisition rate), π (integrase inactivation rate), and *θ* (back-mutation rate for restoration of functional integrase). All other parameter values: as in Figure 2A. Top left: when gene cassette acquisition rate (λ) is too low, no second gene cassette is acquired and I_2_ and M_2_ are not generated (white boxes). Subsequently, all populations are killed following shift from antibiotic A to B. The model predictions from Figure 2A are robust for any given combination of parameter ranges for π, λ, and *θ* depicted with crossed gray boxes. When integrase inactivation rates (π) are too low for the formation of inactive integrases, active integrases only are maintained in the model (open gray boxes).
(TIFF)

Figure S4 Illustration of the model used to determine the existence conditions for Class-1 integrons with a costly, but functional integrase. I_1_ has one-, and I_2_ has two-gene cassettes encoding resistance towards antibiotics A and/or B. M_1_ and M_2_ are integrase defective mutants of I_1_ and I_2_, respectively. Gene-cassette B is acquired at a rate (λ), integrases in populations M_1_ and M_2_ are inactivated at a rate π, and the non-functional integrases are restored by mutation at a rate *θ*.
(TIFF)

Text S1 **Table S1.** Plasmids constructed in this study, **Table S2.** Primers used in this study, **Table S3.** Phenotypic characteristics of strains used in this study, and S1 **References**.
(PDF)
