# Supplementary figures and images for: A Trade-off between the Fitness Cost of Functional Integrases and Long-term Stability of Integrons
Source: PLoS Pathog. 2012 Nov 29;8(11):e1003043. doi: 10.1371/journal.ppat.1003043 (PMC3510236; doi:10.1371/journal.ppat.1003043)

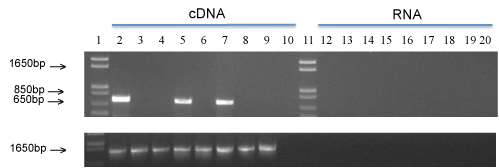

Supplement: Figure S1 — Upper row: RT-PCR with integrase-specific primers (INCINT/IntI1F) confirms expression of the integrase in the constructed strains IVS1 (lane 2), IVS2 (lane 5) and IVS3 (lane 7); for the strains IVS1 intI1::cat (lane 3), IVS1EV-1 (lane 4), IVS2 intI1::nptII sacB (lane 6) and IVS3 intI1::nptII sacB (lane 8) expression of the integrase is below the detection limit. ADP1 was chosen as a negative control for the expression of the integrase (lane 9). RT-PCR does not reveal any amplification with RNA samples in all the strains (lanes 12–19). Lanes 1 and 11- molecular weight marker 1 kb+ DNA-ladder; lanes 10 and 20 - water controls. Bottom row: RT-PCR with 16SrDNA-specific primers (16SF/16SR) was performed to confirm the expression of this gene in all the strains: IVS1, IVS1 intI1::cat, IVS1EV-1, IVS2, IVS2 intI1::nptII sacB, IVS3, IVS3 intI1::nptII sacB, ADP1 (lane 2–9, respectively). RT-PCR with RNA samples does not show amplification of the gene (lanes 12–20). Lane 1- molecular weight marker 1 kb+ DNA-ladder; Lanes 10 and 11 - water controls. (TIFF) [file ppat.1003043.s001.tiff]

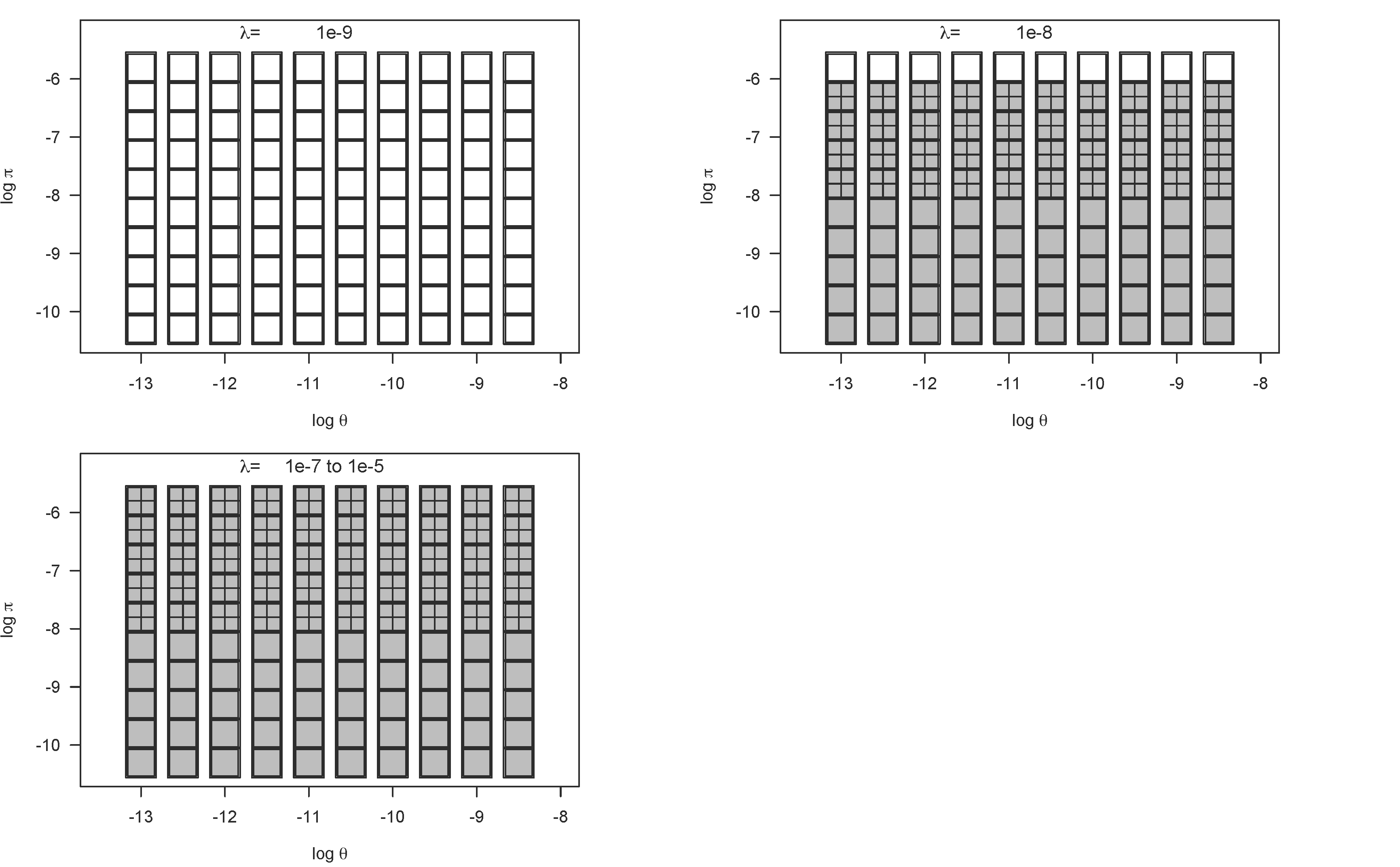

Supplement: Figure S3 — Model predictions for broad ranges of the parameters λ (gene-cassette acquisition rate), π (integrase inactivation rate), and θ (back-mutation rate for restoration of functional integrase). All other parameter values: as in Figure 2A. Top left: when gene cassette acquisition rate (λ) is too low, no second gene cassette is acquired and I2 and M2 are not generated (white boxes). Subsequently, all populations are killed following shift from antibiotic A to B. The model predictions from Figure 2A are robust for any given combination of parameter ranges for π, λ, and θ depicted with crossed gray boxes. When integrase inactivation rates (π) are too low for the formation of inactive integrases, active integrases only are maintained in the model (open gray boxes). (TIFF) [file ppat.1003043.s003.tiff]
