# Supplementary material for: A Trade-off between the Fitness Cost of Functional Integrases and Long-term Stability of Integrons
Source: PLoS Pathog. 2012 Nov 29;8(11):e1003043. doi: 10.1371/journal.ppat.1003043 (PMC3510236; doi:10.1371/journal.ppat.1003043)
Supplement: Text S1 — Table S1. Plasmids constructed in this study, Table S2. Primers used in this study, Table S3. Phenotypic characteristics of strains used in this study, and S1 References. (PDF) [file ppat.1003043.s005.pdf]

Supporting text S1

Table S1. Plasmids constructed in this study

**Plasmid**	**Relevant genotype and phenotypes**	**Size**
pTM2	Contains *cat*, *bla* and an ACIAD3309::(*nptII sacB*) allele	8546 bp
pTM4	Contains *cat* and an ACIAD3309::*Sac*I allele	4482 bp
pACYC177-*int-cat*	Contains *bla* and an *'intI1'*::*cat* fusion allele	5742 bp
pACYC177-*int-nptII-sacB*	Contains *bla* and an *'intI1'*::(*nptII sacB*) fusion allele	7370 bp

Table S2: Primers used in this study.

Primer name	Nucleotide Sequence	Reference
Intf2	TCCGCCAGGATTGACTTGCG	This study
OrfRev2	CTAGGCGTTCTGCGATGAAGG	This study
OrfRev3	TCGCGAACCAAGACATCGC	This study
GCS1 RevComp	CGAGGCATTTCTGTCCTGG	This study
5CS’	GCCTGTTCGGTTCGTAAGCT	[1]
3CS’	CGGATGTTGCGATTACTTCG	[1]
INCINTF	TGATGCCTGCTTGTTCTACG	[2]
IntI1F	AGCTTACGAACCGAACAGGC	This study
Sul-out-F2	CGGAACTTCACGCGATCGGCAATGG	This study
Orf5-out-F2	CCATGCCGCGCGAACGCAGGGGTGC	[3]
UpF2	AGTCTTCCCCAGCCTGCA	This study
DownR	CGTCTTCAAGCATTTGAAGG	This study
ACIAD3309-up-F	GTAAGTCTTCCCCAGCCTGCACG	This study
ACIAD3309-up-R	AGAGCTCAAACCGCATATTATTTCCGTTAAAACACG	This study
ACIAD3309-down-F	CTTCTAGA**G**CTCTGTCGGTGCAGCGTATAGTCTATCC	This study
ACIAD3309-down-R	AACTTGAGCGTCTTCAAGCATTTGAAGG	This study
aacC1-F2	TACGTGCAAGCAGATTACGG	[4]
aacC1-orfP-R	ATCTCGGCTTGAACGAATTG	[4]
aadBF	CAATGCCTGACGATGCGTGG	This study
aadBR	CGCCAACTATTGCGATAACA	This study
OXA305F	GGAGCAGCAACGATGTTACG	[5]
OXA303R	CGACTTGATTGAAGGGTTGG	[5]
16SF	GATCATGGCTCAGATTGAACG	This study
16SR	ACGACTTGACCCCAGTCATC	This study

Table S3. Phenotypic characteristics of strains used in this study.

Minimal inhibitory concentration (MIC)							
**Strain**	**Km**	**SP**	**ST**	**GM**	**AP**	**Sucrose#**	**Ref**
ADP1	0.5	0.1	0.125	0.25	0.125	R	[6]
A. baumannii 064	>256	48	96	256	96	R	[2]
IVS1	24	*	*	*	*	R	TS
IVS1 *intI1*::*cat*	24	*	*	*	*	R	TS
$IVS1_{EV-1}$	24	*	*	*	*	R	TS
$IVS1_{EV-2}$	24	*	*	*	*	R	TS
$IVS1_{EV-3}$	24	*	*	*	*	R	TS
IVS4	24	*	*	*	*	S	TS
S. enterica serovar Typhimurium 490	*	96	8	0.38	>256	R	[2]
IVS2	*	16	6	0.125	16	R	TS
IVS2 *intI1*::*nptII sacB*	16	16	6	0.125	16	S	TS
A. baumannii 47-42	>256	>1024	64	>256	>256	R	[4]
IVS3	0.5	16	8	2	*	R	TS
IVS3 *intI1*::*nptII sacB*	0.5	16	8	2	*	S	TS

KM-kanamycin, SP-spectinomycin, ST-streptomycin, GM-gentamycin, AP-ampicillin
MIC is not applicable
[7,8]
TS: This study

References

1. Toleman MA, Vinodh H, Sekar U, Kamat V, Walsh TR (2007) blaVIM-2-harboring integrons isolated in India, Russia, and the United States arise from an ancestral class 1 integron predating the formation of the 3' conserved sequence. Antimicrob Agents Chemother 51: 2636-2638.

2. Domingues S, Harms K, Fricke WF, Johnsen PJ, da Silva GJ, et al. (2012) Natural Transformation Facilitates Transfer of Transposons, Integrons and Gene Cassettes between Bacterial Species. PLoS Pathogens 8: e1002837.

3. Fournier PE, Vallenet D, Barbe V, Audic S, Ogata H, et al. (2006) Comparative genomics of multidrug resistance in *Acinetobacter baumannii*. PLoS Genetics 2: e7.

4. Karah N, Haldorsen B, Hermansen NO, Tveten Y, Ragnhildstveit E, et al. (2011) Emergence of OXA-carbapenemase- and 16S rRNA methylase-producing international clones of *Acinetobacter baumannii* in Norway. J Med Microbiol 60: 515-521.

5. Hanson ND, Moland ES, Hossain A, Neville SA, Gosbell IB, et al. (2002) Unusual *Salmonella enterica* serotype *Typhimurium* isolate producing CMY-7, SHV-9 and OXA-30 beta-lactamases. J Antimicrob Chemother 49: 1011-1014.

6. Barbe V, Vallenet D, Fonknechten N, Kreimeyer A, Oztas S, et al. (2004) Unique features revealed by the genome sequence of *Acinetobacter sp*. ADP1, a versatile and naturally transformation competent bacterium. Nucleic Acids Res 32: 5766-5779.

7. Dedonder R (1966) Levansucrose from *Bacillus subtilis*. Methods Enzymol 8: 500-505.

8. Harms K, de Vries J, Wackernagel W (2007) A double kill gene cassette for the positive selection of transforming non-selective DNA segments in *Acinetobacter baylyi* BD413. J. Microbiol Met 69: 107-115.
